# Pain Management During Adult Laparoscopic Appendectomy: A Systematic Review

**DOI:** 10.7759/cureus.52037

**Published:** 2024-01-10

**Authors:** Stefan J Biput, Ethan Slouha, Jheanelle A Gregory, Brandon Krumbach, Lucy A Clunes, Theofanis F Kollias

**Affiliations:** 1 Medicine, St. George's University School of Medicine, St. George's, GRD; 2 Anatomical Sciences, St. George's University School of Medicine, St. George's, GRD; 3 Anesthesia, St. George's University School of Medicine, St. George's, GRD; 4 Pharmacology, St. George's University School of Medicine, St. George's, GRD; 5 Microbiology, Immunology and Pharmacology, St. George's University School of Medicine, St. George's, GRD

**Keywords:** opioids, appendicitis, pain management, anesthesia, laparoscopic appendectomy

## Abstract

The opioid epidemic has become a critical public health issue, driven by the widespread distribution and misuse of prescription opioids. This paper investigates analgesic management in the context of laparoscopic appendectomy (LA) as an alternative to open appendectomy, aiming to reduce the reliance on opioids for postoperative pain control. A comprehensive literature review was conducted from January 1, 2003, to November 1, 2023, utilizing PubMed, ProQuest, and ScienceDirect databases. The search focused on peer-reviewed experimental and observational studies involving adults (18 years and older) undergoing LA. The original search resulted in 18,258 publications, which were then screened using PRISMA guidelines. Among the filtered 18 studies included for analysis and review, the transition from open to LA demonstrated a consistent decrease in postoperative pain, leading to a reduced need for opioid prescriptions. Analgesic strategies included the use of local anesthetics (lidocaine, bupivacaine, ropivacaine), spinal/epidural anesthesia, nerve blocks, and a multimodal approach with NSAIDs and acetaminophen. Studies demonstrated the efficacy of local anesthetics in reducing postoperative pain, prompting a shift toward non-opioid analgesics. The use of spinal/epidural anesthesia and nerve blocks further supported the trend of minimizing opioid prescriptions. While some variations in anesthetic approaches existed, overall, patients undergoing LA required fewer opioid doses, reflecting a positive shift in postoperative pain management. Patients undergoing LA experienced lower rates of readmission, reduced post-operative pain, better cosmetic outcomes, and shorter recovery times, contributing to a diminished demand for opioid medications. This review underscores the potential for non-opioid analgesic strategies in surgical contexts, aligning with the broader imperative to address the opioid epidemic and promote safer and more sustainable pain management practices.

## Introduction and background

Opioid epidemic

The opioid epidemic arose due to the increased distribution and misuse of prescription opioids, leading to higher rates of abuse [1]. One theory of origin traces back to the late 1990s when the American Pain Society decided to recognize pain as the fifth vital sign and consequently decided to liberalize the prescription of opioids to patients [1,2]. Based on this initiative, it was seen to be inhumane if opioids were not prescribed to patients in pain, which could have resulted in litigation for under-treatment of pain [2]. Therefore, medical physicians and pain management teams subsequently relied more heavily on opioids for pain treatment. However, due to the incorrect use of prescription opioids, there were drastic increases in the mortality rates due to opioid overdose. According to the National Vital Statistics System mortality statistics from the Centre for Disease Control and Prevention (CDC), there has been a proportionate quadruple increase in the prescription of opioids as well as mortality in both men and women [2]. Consequently, the opioid epidemic was declared a public health emergency on October 16, 2017, and immediate solutions were needed [2].

To try and immediately solve the opioid epidemic, researchers developed abused-deterrent modified opioids to prevent opioid misuse and overdose. Opioids were modified to have tamper-resistant packaging to include the opioid and a low-dose opioid antagonist in the formulation; these fell under the initiative Risk Evaluations and Mitigation Strategies (REMS) [2]. Along with pharmacological therapies, physicians are trained to identify patients who may benefit more from non-pharmacological treatment. According to Jones et al., physicians should be trained to evaluate the psychosocial risk factors affecting self-reporting pain, set realistic goals for treatment plans, emphasize impairment of physical function, and promote the use of non-pharmacologic pain treatments [2]. As expected, by incorporating this approach, there has been a decrease in the number of opioids prescribed, further resulting in a decrease in the number of opioid overdose mortalities. Initiatives such as the Prescription Drug Monitoring Programs (PDMPs) and the National All Schedules Prescription Electronic Reporting Act (NASPER) have reduced opioid prescriptions by 8% and prescription opioid overdose death rates by 12% [1]. However, there is no doubt that some patients ultimately require opioids. Physicians have access to appropriate data so that they can be knowledgeable to identify the barrier between preventing opioid misuse and beneficial opioid treatment for medical interventions.

Appendectomy

Currently, early treatment of appendicitis is mainly focused on surgical methods via an appendectomy procedure. More than 300,000 appendectomies are performed annually in the USA [3,4]. Abraham Groves performed the first elective appendectomy in 1883 [5]. Charles McBurney described an incision parallel to the right rectus muscle oblique at approximately 1-4 inches, now known as the “McBurney-McArthur muscle-splitting incision” [5,6]. This incision is associated with the lowest mortality, provides easy access to the appendix, facilitates drain placement, and can be closed without risking herniation [5].

However, in 1983, about a century later, Semm introduced laparoscopic appendectomy as an alternative to open appendectomy to treat appendicitis [5,6]. In laparoscopic appendectomy, a total of three incisions are made to facilitate the trocars. The first incision is 5-10 mm at the umbilicus for the permanent metallic trocar, followed by two bilaterally and medially to the epigastric vessels in the suprapubic region for alternative port sites [7]. Patients who underwent laparoscopic appendectomy had lower rates of readmission, lower costs, reduced post-operative pain, better cosmetic appearance, lower risk of infections, and shorter recovery time compared to open appendectomy [3-6,8]. As a result of the decreased post-operative pain and shorter hospital stays, it can be deduced that there was a decrease in the prescription of analgesic drugs for patients who underwent laparoscopic appendectomy.

Aim

The misuse and abuse of opioids has become a public health concern due to the increasing number of mortalities from opioids, particularly prescription opioids. One of the main causes is attributable to physicians being heavily reliant on opioids to manage their patient’s pain levels following surgical procedures. Due to increasing research and advancements in medicine, surgical operations have been modified to enhance better clinical outcomes, for example, open appendectomy versus laparoscopic appendectomy. As a result of advancement, fewer postoperative complications have arisen, subsequently implying that there should be less need for prescription opioid pain control or the use of alternate medication. Therefore, this paper aims to determine the pattern of analgesic use in patients undergoing laparoscopic appendectomy, as well as to review the necessity to use opioid medications throughout surgery whilst ensuring the patient has a satisfactory pain level.

## Review

Methods

An exhaustive and meticulous literature search was done using PubMed, ProQuest, and ScienceDirect databases from January 1, 2003, to November 1, 2023. Keywords included “analgesics and laparoscopic appendectomy” and “pain management and laparoscopic appendectomy.” The electronic search focused mainly on peer-reviewed, experimental, and observational publications on adults 18 years and older. Publications written in a language other than English published prior to 2003, and duplicates were excluded from the eligibility review. Once the publications were acquired, four independent co-authors examined the information and compiled and compared the results. The publications found in the search were examined based on the full-text accessibility, study type, title, age of participants, and abstract. The original search of the three databases resulted in 18,258 publications. Selected publications were removed based on keyword specifics, age range, and the overview provided by the abstracts. A total of 18 publications were found to be eligible, and that covered the intent of this paper according to the following criteria.

Inclusion Criteria

The inclusion criteria for this study comprised publications conducted on humans, published between 2003 and 2023, focused on all types of analgesics used with LA, full-text availability, and were peer-reviewed experimental or observational studies.

Exclusion Criteria

**Figure 1:**
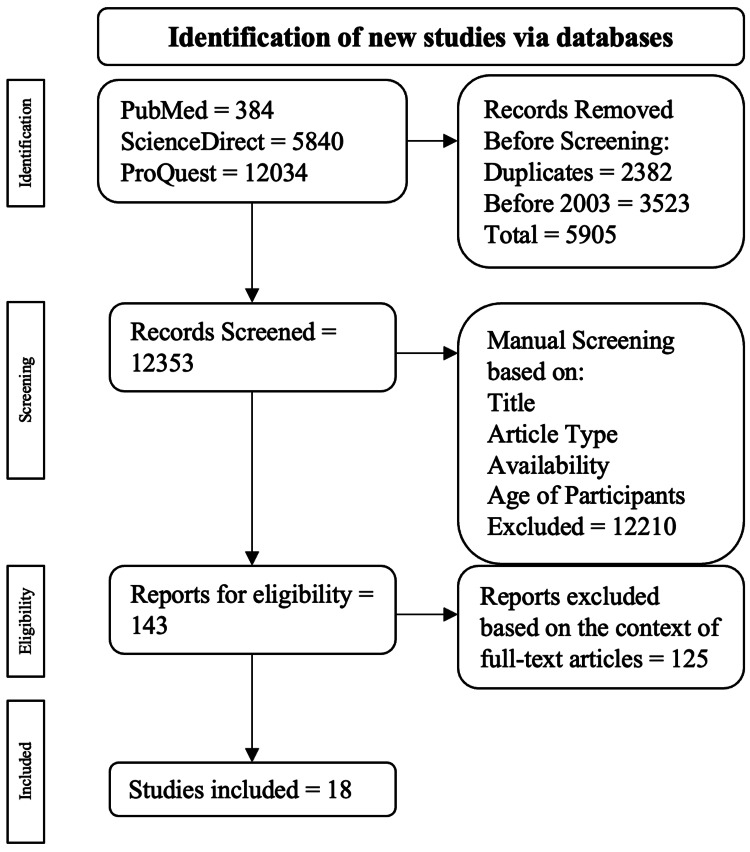
Visual representation of the inclusion and exclusion criteria for filtering articles. The pathway used for the inclusion and exclusion criteria came from the PRISMA review [9].

Exclusion criteria comprised any publications that were meta-analyses, case reports, case series, and narrative reviews. All non-full-text publications and duplicates were also excluded. The inclusion and exclusion methods for this publication are drawn out in Figure 1.

Bias

All studies included in this publication were assessed for bias. The studies had an overall minimal risk of bias due to incorporating both observational and experimental study standards. Each publication included was individually assessed using the GRADE (grading of recommendation, assessments, development, and evaluation) scale. The GRADE tool assessed each article’s imprecision, indirectness, and publications to determine the risk of bias.

Results

A total of 18,258 publications were found: 384 were from PubMed, 5,840 were from ScienceDirect, and 12,034 were from ProQuest. Among those excluded, 2,382 were duplicates, and 3,523 were published before 2003. This resulted in 5,905 publications being excluded during the automatic screening process, leading to 12,353 publications for manual selection. Publications were manually screened based on title, article type, availability, and age of participants, resulting in 143 being evaluated for eligibility via full-text analysis. Ultimately, 18 articles were used.

Due to the advent of laparoscopic appendectomy, it was found that overall, there was a decrease in the postoperative pain experienced by patients compared to open appendectomy, which was used previously. Therefore, there should be a decreased need for opioid prescriptions to help in managing the opioid epidemic. The use of anesthetics such as lidocaine, bupivacaine, and ropivacaine, both systemically and locally in wound infiltration, spinal/epidural anesthesia, and nerve blocks, has been found to reduce the pain experienced following the operation, subsequently requiring less prescribed opioid requirements. Standardized postoperative pain management drugs included NSAIDs, acetaminophen, and opioids. The majority of patients received baseline NSAIDs or acetaminophen, only requiring opioids to treat breakthrough pain. A trend was identified throughout the articles whereby patients experienced comparative pain satisfaction levels for those who used opioids compared to those who did not. Additionally, it was discovered that patients who received fewer prescribed opioids upon discharge were less likely to use them compared to those prescribed a larger number of opioids. Publications used in this review are summarized in Table 1.

**Table 1:** Summary of articles used in this review as stated in the PRISMA guidelines [9]. LA - Laparoscopic Appendectomy; PCA - Patient Controlled Analgesia; TAP - Transabdominal Plane; TSA - Trocar Site Anesthesia; OA - Open Appendectomy; IV - Intravenous; IP - Intraperitoneal; QL - Lateral Quadratus; LB - Lumborum Block

	Author	Country	Design & Study Population	Findings	Conclusion
1	Biondi et al., 2016 [10]	Italy	Retrospective Cohort Study (n = 593)	LA was significantly associated with a shorter hospital stay, quicker return to daily activities, and less analgesia. The number of complications and operation time was significantly decreased in LA patients. The cost of treatment, however, was higher in patients in the LA group.	Ultimately, patients who undergo LA require less pain medication, quicker recovery, shorter hospital stays, and fewer complications, but the procedure cost is higher.
2	Neethirajan et al., 2020 [11]	India	Experimental Study (n = 60)	The average duration of analgesia was greater in patients receiving both bupivacaine and dexmedetomidine. These patients also required significantly fewer rescue analgesics.	Bupivacaine and dexmedetomidine together significantly decreased pain and the need for rescue analgesics.
3	Sertcakacilar et al., 2022 [12]	Turkey	Experimental Study (n = 136)	There was no statistically significant evidence showing a difference between the groups in pain control regarding intraoperative remifentanil consumption, postoperative total opioid consumption, and pain scores.	Ultrasound-guided QL LB may aid in analgesic efficacy in patients having an LA and can be considered in multimodal analgesic pain control, but no statistically significant evidence favors the TAP over QL blocks.
4	Thanapal et al., 2014 [13]	Malaysia	Experimental Study (n = 120)	Postoperative patients in the placebo group required significantly more analgesia compared to ropivacaine and levobupivacaine. Postoperative cortisol levels were significantly higher in the placebo group than in the experimental group.	Intraperitoneal injection of local anesthetic in LA significantly improved patients' pain control postoperative and reduced serum cortisol levels, reducing metabolic stress response to the procedure.
5	Erdem et al., 2018 [14]	Turkey	Prospective Randomized Study (n = 50)	Surgical pain after surgery was significantly lower in the spinal anesthesia group (p < 0.001). In the spinal anesthesia group, intraoperative issues included shoulder pain, abdominal discomfort/pain, anxiety, and hypotension. Right shoulder pain after surgery was significantly higher in the general anesthesia group compared to the spinal anesthesia group.	Spinal/epidural anesthesia is safe and effective for healthy ASA I patients undergoing LA, offering benefits such as reduced postoperative pain, less postoperative nausea and vomiting, and reduced shoulder pain compared to general anesthesia.
6	Huang et al, 2019 [15]	Australia	Randomized Control Trial (n = 86)	In the early postoperative phase (0-6 hours), there was a notable decrease in the number of times patients pressed the PCA button in the ropivacaine group compared to the normal saline group (16 times versus 24 times).	Ropivacaine administered intraperitoneally provides pain relief for patients for up to 6 hours after undergoing emergency LA.
7	Kang et al., 2010 [16]	Korea	Experimental Study (n = 63)	The instillation group received 2mg/kg ropivacaine and had decreased visual analog pain scores, fentanyl usage, and usage of the patient-controlled analgesia system during postoperative compared to group C.	By using ropivacaine intraperitoneally, there was reduced postoperative pain after LA, as well as a reduced patient requirement for postoperative opioids.
8	Kim et al., 2011 [17]	Korea	Experimental Study (n = 68)	It was found that groups IV and IP had reduced pain scores and fentanyl consumption compared to group C (received IV and intraperitoneal saline), as well as decreased shoulder tip pain and postoperative nausea and vomiting. No significant differences were found between groups IP and IV.	IV lidocaine and intraperitoneal instillation have similar effects for decreasing pain and fentanyl usage in patients who underwent LA. IV lidocaine is preferred as it is more convenient and easily administered compared to IP instillation.
9	Tupper-Carey et al., 2017 [18]	Singapore	Experimental Study (n = 58)	Mean postoperative morphine consumption was reduced in patients receiving supplemental TAP block. The length of stay was shorter for the TAP intervention group.	TAP block did not significantly improve postoperative analgesic outcomes.
10	Molfino et al., 2018 [19]	Italy	Prospective Case-Control Study (n = 133)	There was no significant difference in pain following awakening between TAP or TSA, but a significant decrease compared to those without pre-incisional treatment.	TSA and TAP blocks drastically reduced pain levels from awakening at all times.
11	Jun et al., 2014 [20]	South Korea	Prospective Observational Study (n = 26)	All surgeries were successfully completed laparoscopically without any need for conversion to general anesthesia or open surgery. Seventeen patients (65.4%) received additional fentanyl or ketamine injections, and seven patients (26.9%) experienced bradycardia.	Combining spinal anesthesia with dexmedetomidine infusion is feasible for LA but requires additional analgesia, sedation, and vigilant monitoring to prevent bradycardia and ensure a successful outcome.
12	Waddimba et al., 2022 [21]	USA	Retrospective Cohort Study (n = 155)	Postoperative pain scores were equivalent across all cohorts. Postoperative opioid use was higher in the bupivacaine group vs the liposomal bupivacaine group.	Analgesia with liposomal bupivacaine during LA can reduce inpatient opioid use without increasing post-op pain scores.
13	Sevensma et al., 2019 [22]	USA	Experimental Study (n = 101)	Pain scores at the 1-hour mark were significantly improved in the bupivacaine group over the saline group. Pain scores were improved but not significantly in the 2,4, and 12-hour mark. Length of stay was significantly decreased in the bupivacaine group. Postoperative opioid dose use was significant.	IP bupivacaine injected before close significantly decreased pain scores compared to the saline-injected control group. Postoperative opioid use was significantly decreased in the bupivacaine group, and length of stay was decreased between the groups but not statistically significant.
14	Rao et al., 2022 [23]	Singapore	Retrospective Cohort Study (n = 201)	Comparing LA patients to OA patients, LA patients recorded less pain. The length of stay for LA was significantly less, with a mean of 3.09 days compared to OA's mean of 6.93 days. Almost 87% of patients did not complete the prescribed analgesics and claimed the hospital leave was more than they needed.	A significant number of the LA patients did not need 2 weeks of analgesics, which was the protocol for this medical center. Also, the length of stay in the hospital can be reduced for a quicker return to work.
15	Geetha et al., 2009 [24]	India	Prospective Cohort Study (n = 200)	LA had a better outcome than OA regarding wound infection rates, the time taken to resume oral feeding, postoperative pain, and lesser use of analgesics, as well as postoperative hospital stay and time taken to return to normal activities. However, LA was more expensive and had a longer operating time than OA.	LA was deemed to have better recovery periods for patients in multiple aspects, however, it was more expensive and had a longer duration of operating time compared to OA.
16	Choi et al., 2017 [25]	Korea	Retrospective Cohort Study (n = 753)	There was reduced pain and rescue analgesic use in patients undergoing single-incision LA compared to conventional LA on the day of the surgery.	SILA leads to reduced pain and medication use on the day of the surgery compared to CLA.
17	Feinberg et al., 2021 [26]	Canada	Experimental Study (n = 295)	In the first phase of the study, 20 opioid tablets were given, and between the LA and laparoscopic cholecystectomy patients, the median number of pills consumed was 2, with a 95% satisfaction rating. In the second phase, only 10 pills were given, and the median number of opioid pills filled were significantly decrease with no consumption, and there was no difference in the reported satisfaction of pain control.	Patients are generally prescribed an excess of opioid medications, and with the advancement of laparoscopic surgery the prescribed amount can significantly decrease as the use has significantly decreased.
18	Hayes et al., 2022 [27]	USA	Experimental Study (n = 1785)	1785 LAs were done, with 23.6 doses of opioids given in the control period compared to 14.2 doses given in the intervention period. There was an average of 40% decrease in the prescription of opioids.	By evaluating the amount of prescribed opioids to patients who underwent la, this data can be used by surgeons to control how much they prescribe to their patients.

Discussion

Pain Management Throughout the Laparoscopic Appendectomy

Anesthesiologists have different preferences in their choice of drug use for inducing and maintaining anesthesia for patients undergoing laparoscopic appendectomy. Furthermore, some physicians chose to pre-medicate their patients with individualized doses of midazolam based on their weight [10-13]. For anesthesia induction, patients were administered a combination of 1-3 mg/kg propofol, 1-2 μg/kg fentanyl, 0.1 mg/kg vecuronium, 1-1.5 mg/kg suxamethonium, and/or 1-1.5 mg/kg desflurane in oxygen-enriched air mixture, and this was maintained typically with sevoflurane 1-3% with oxygen [11-18]. Alternatively, another study gave their patients 5 mg/kg thiopental and 0.6 mg/kg rocuronium intravenously for anesthesia induction and tracked if they altered pain following surgery [16,17].

After inducing and maintaining the patient under anesthesia, some studies started to alter their use of anesthetics to improve the pain experienced following surgery. Kim et al. assessed the use of lidocaine to reduce postoperative pain, giving 0.55 mL/kg of 1% lidocaine IV and 1.75 mL/kg of 2% lidocaine for wound infiltration. Right after the pneumoperitoneum was made and 10 minutes before surgery, patients received an intraperitoneal instillation of lidocaine or placebo solution spray on the upper surface of the liver under the right subdiaphragmatic space and the left subdiaphragmatic space [17]. In another study, 50 mL of ropivacaine or placebo solution was sprayed on the upper surface of the liver and the right subdiaphragmatic space [16]. After creating the pneumoperitoneum, one group received ropivacaine, and the other received placebo solutions [16]. Infiltration of the wound was also a choice for preoperative pain management. It was done by using levobupivacaine or ropivacaine in the skin and pre-peritoneal space before introducing the ports and then again after the third port was introduced [13,15,19].

Other methods of pain management were through spinal/epidural anesthesia via a needle-through-needle method using 2 mL of 2% lidocaine at the needle entry site and then 10 mg of hyperbaric 0.5% bupivacaine and 10 μg of fentanyl [14,20]. The epidural anesthesia itself was done using 10 0.5% bupivacaine, 25 μg fentanyl, and 5mL isotonic saline via the catheter [14]. A different sample of patients received anesthesia via an ultrasound-guided transabdominal plane (TAP) block [12,18,19]. Two different techniques were noted: TAP or quadratus lumborum (QL). Both studies performing the TAP block did so by ultrasound-guided; however, one study injected bupivacaine, while the other injected ropivacaine bilaterally between the internal oblique muscles and the border of the transverse abdominal muscle [12,18]. For the QL block, bupivacaine was also injected bilaterally between the layer of the thoracolumbar fascia and the QL muscle [12]. One study, however, did not specify the fascia block used but injected liposomal bupivacaine as their experimental group with epinephrine to compare pain control to standard bupivacaine [21].

During the laparoscopic appendectomy, patients were given additional medication for pain control. After administering IV 1.5 mg/kg lidocaine, some patients were continuously infused with IV lidocaine at 2 mg/kg/h [17]. Other physicians decided to use IV 1 g of paracetamol, parecoxib, dexamethasone, granisetron, and ketorolac [15,19]. For additional pain relief, IV fentanyl was given alone or along with ketamine [18,20]. At the end of the procedure, one study instilled blinded solutions using either a placebo of 0.9% saline or 0.2% ropivacaine, while another administered bupivacaine [15,22].

Further pain management requires immediate analgesics through a standard multimodal analgesic approach for postoperative pain [12,23]. In some studies, each group received 30 mg IV tenoxicam or etoricoxib NSAID every 12 hours, whereas some patients received paracetamol every 6-8 hours with ketorolac given on demand [12,18,19,24]. Geetha et al. observed that LA required significantly less NSAID dosing than open appendectomies [24]. Common opioids that are prescribed are tramadol, morphine, and fentanyl, which are given either initially or for pain rescue [12-18,25]. It was observed that several physicians opted to use patient-controlled analgesia (PCA) for postoperative pain relief following the laparoscopic appendectomy [12,13,15-18,25]. Although these studies all used PCA for post-op analgesia, they each had their preferred medication used along with criteria for measuring pain and termination of the treatment. PCA morphine in one study was used for at least 12 hours for all patients where termination was based on predefined criteria of pain less than or equal to 4 at rest and movement [18]. Another preferred regimen was a bolus of 5 mg/mL tramadol hydrochloride, a demand dose of 10 mg with a lock time of 15 minutes, and a maximum daily dose of 400 mg without basal infusion [12].

Some physicians had a preference for using fentanyl instead for their PCA, along with additional pain medication, such as ketorolac, acupan, ramosetron, paracetamol, and celecoxib [15,25]. However, even with PCA, if patients still had persistent pain, an additional 50 μg fentanyl was given IV or a rescue analgesic of 1 mg IV meperidine [16,17]. Once PCA was discontinued, patients were prescribed rescue analgesics if their pain exceeded 4 or if the pain team thought it necessary based on clinical grounds [18]. Some patients were given tramadol immediate release (IR) and/or oxycodone IR for rescue analgesia [15]. Post-operative nausea and vomiting were controlled using IV ondansetron 4 mg [16]. In one study, each discharged patient received hydrocodone 5 mg/acetaminophen 325 mg until pain subsided [22].

Outcomes Following Laparoscopic Appendectomy

A considerable number of studies have shown that laparoscopic appendectomy is a better option than open appendectomy regarding length of stay, early return to work, overall analgesic requirements, postoperative pain scores, surgical complications, and infections [10,23-25]. In patients who underwent single port LA the use of fentanyl-based pain pumps was significantly lower in patients who underwent single-port ILA [25]. For patients who received intra-peritoneal lidocaine, bupivacaine, levobupivacaine, or ropivacaine, there was a significant reduction in pain scores postoperative, along with decreased fentanyl and PCA usage compared to patients who received saline instead [13,15-17,22]. Two studies found that the patients who received ropivacaine or levobupivacaine had decreased total postoperative analgesia needed compared to the normal saline group in the first six hours [13,15]. However, no significant disparities were seen between the two groups within 6-16 hours post-operative [15]. Subsequently, another study stated that patients who received ropivacaine had significantly reduced pain scores at all times, except at 24 hours, compared to the placebo group [16]. Sevensma et al. also observed that bupivacaine use was associated with a decrease in the length of hospital stay [22].

There were statistically significant lower postoperative pain scores immediately after the operation for patients who underwent spinal anesthesia, compared to general anesthesia, with patients requiring tramadol infusion at zero and six hours post-op [14,20]. In one of the groups for spinal anesthesia, 65.4% received additional fentanyl or ketamine injections, with 26.9% experiencing bradycardia [20]. Therefore, the study concluded that a combination of spinal anesthesia with dexmedetomidine infusion was feasible for laparoscopic appendectomy but required additional analgesia, sedation, and vigilant monitoring to prevent bradycardia.

Additionally, for patients that underwent fascial block with bupivacaine HCL (BH) and BH diluted liposomal bupivacaine (LB), there were significant differences for inpatient opioid use as well as lower frequency of usage between the groups, but there were no significant differences in the pain scores between the groups [21]. A study also reported this finding comparing wound infiltrations and TAP block with levobupivacaine [19]. Moreover, it was found that patients who received pre-incisional anesthesia required fewer rescue doses of ketorolac, reduced length of stay, and statistically significantly decreased pain levels with both wound infiltration and TAP block compared to patients who did not receive pre-incisional anesthesia [19]. Patients who did receive TAP block or QL block using ropivacaine or bupivacaine had lower mean pain score values. Still, statistical significance varied between studies, as well as reduced opioid usage [12,18]. With regards to TAP blocks and QL blocks, both provided post-operative pain control for laparoscopic appendectomy. Still, TAP blocks were preferred due to the increased ease of ultrasound guidance and more clinical experience, as no complications such as vascular puncture, anesthetic toxicity, and hematoma were reported [12].

There was variation between the studies with the reported adverse effects. For patients who received general anesthesia, the common complaint was postoperative nausea and vomiting, which was controlled using IV ondansetron 4 mg [14,16]. Shoulder pain was the most common adverse effect after surgery [14,15,17,20]. Secondly, patients who received spinal anesthesia reported headaches, urinary retention, hypotension, bradycardia, and abdominal pain [11,14,20]. Hypotension was treated using 10-15 mg ephedrine, bradycardia was treated with 0.5 mg atropine, and abdominal pain was treated with injections of fentanyl with or without ketamine [20].

Rao et al. reported their follow-up findings with telephone interviews. It stated that 59% of patients consumed their analgesics for less than five days, and almost 83% did not take them for more than seven days [23]. Still, there was no statistical significance between analgesic requirements and leave duration amongst various ethnic groups [23]. In addition, in comparing patients who were prescribed 10 opioid pills versus those prescribed 20 opioid pills, there was no difference in pain scores or patient satisfaction between the groups [14,26]. There was, however, a statistically positive association between the number of pills prescribed and the number consumed [14,26]. As a result, despite intervention and feedback, 46% of laparoscopic appendectomy patients still received more than the recommended number of opioids post-op [27].

Some limitations of this study were the lack of publications focusing on adult LA, an array of analgesic techniques, and a lack of clarification on the type of LA used in all surgeries. Only 18 publications focused on the topic, which reduces the strength of their report and the conclusion deduced, but there is complete consistency in that LA had reduced pain levels than open appendectomy. While it is great that there is an array of studies evaluating the difference in pain management for LA, there was a lack of replication in pain management to show the reproducibility of the results. This means their findings should be concluded cautiously, but the intent to reduce the need for opioids and pain following surgery is crucial. Lastly, one study presented that conventional versus single port LA have different pain levels following surgery, which indicates that it may be important to consider the type of LA performed. While publications focused on operative treatment, retrospective studies did not include the type of LA performed. Nonetheless, this review creates a ground for further research to be performed to reduce the use of opioids, either due to improving pain management or simply educating surgeons that there’s already a reduced need for opioid use.

## Conclusions

The opioid epidemic is continuing to rise, drastically increasing over the past decade, and is associated with a high incidence of deaths due to overdose. There is a need to address this crisis, and one starting point is focusing on how individuals get addicted. Incidentally, a good portion of opioid abusers receive their first dose from physicians to treat real pain. The goal is to address this by educating physicians that non-opioid analgesics may be able to alleviate pain almost, as well as opioids in non-complicated cases. Appendicitis is a relatively common condition that is caused by blockage of the appendix, usually by a fecalith, and then becomes inflamed. Appendicitis can require surgery to cure the condition, and after surgery, there is associated pain that has been historically treated with opioids before, during, and after the surgery. Appendectomies have transitioned from an open surgical procedure to a laparoscopic procedure, requiring less trauma to the individual. Therefore, there should be a need for significantly fewer pain medications, specifically opioid use following surgery.

Studies have evaluated pain management used throughout the stages of appendectomies. Induction and maintenance of anesthesia are relatively standard, individualizing some medications based on weight. However, studies have also evaluated different anesthetics used for wound infiltration before port insertion, such as lidocaine, bupivacaine, and ropivacaine. It was found that the addition of these medications reduces the pain experienced following the surgery, resulting in the need for less analgesia, specifically opioids. Other studies have evaluated spinal anesthesia or epidural blocks with the use of fentanyl and also found that this leads to reduced pain scores and the need for opioids following surgery. This is a different type of opioid use, as the favored euphoric effects do not occur as this method essentially paralyzes the nerves. Another anesthetic procedure is the use of TAP and QL blocks, which were also successful in reducing postoperative pain and the need for analgesics. Current protocols for pain management after surgery include a mix of NSAIDs, acetaminophen, and opioids, with more patients receiving baseline NSAIDs or acetaminophen and opioids for breakthrough pain. However, some surgeons put patients on PCA pumps with baseline fentanyl or tramadol to treat pain despite this being shown to be unnecessary. Studies evaluating follow-up satisfaction and opioid use found similar satisfaction of pain management without opioids compared to with opioids. In patients receiving fewer opioid medications after discharge, they were less likely to use the opioids. With the rise in the opioid epidemic, there needs to be a limitation towards the application of opioids where other alternatives that prove to be just effective are available, which can be accomplished by controlling the dispense of opioids in healthcare. Research shows there is a reduced need for opioids, and further research focused on improving postoperative pain shows that there will be an even further reduction in opioid use. These studies have shown success in such reduction by addressing wound infiltration, spinal/epidural anesthesia, and nerve blocks. Further studies should focus more on these approaches as limited research was found regarding each method. As a better understanding is developed concerning these methods, research should then compare each method to determine the optimal analgesic approach throughout the treatment of appendicitis. While these are crucial, education of the prescribing physicians needs to be widely employed so that they know opioids should be the last resort and not a first choice when managing pain.
